# M. Civiale on Catarrh of the Bladder

**Published:** 1842-04-30

**Authors:** 


*Catarrh of the Bladder.*—In the last volume of his excellent treatise on diseases of the genito-urinary organs, M. Civiale makes some observations on the treatment of catarrh of the bladder by injections, which are worthy of the attention of practitioners. M. Civiale assures us that he had obtained the best results from this mode of treatment in cases of chronic catarrh. The object of injections is to remove the urinary deposits from the bladder, modify the sensibility of the organ, and restore its contractile power.

Simple water should be used at the commencement, either tepid or cold, according to circumstances, and the injections are to be continued as long as the urine contains any deposit, and the bladder remains weak.

Immediately after a natural evacuation of the urine, a catheter should be passed into the bladder to ascertain whether all the urine has been expelled, and whether the portion which may remain contains any deposits or not. This done, some tepid water must be slowly injected into the bladder, and after a very short sojourn there, allowed to flow away. The same operation must be repeated every day, unless the urethra and bladder show some signs of irritability, when it may be suspended for a day or two. Generally speaking, in from three to eight days the patient is able to make water more easily, and the urine contains less sediment.

Two injections are now made, one upon the other. While the first is flowing away, the syringe is filled again, and a second injection thrown into the bladder. Should the expulsive power of the organ be extremely feeble, three to five injections may be thus made, one on the other, and the temperature of the fluid gradually reduced. The best indication of the number of injections to be employed, and the temperature of the water, is the manner in which the fluid escapes from the bladder. At first it merely dribbles away, but as soon as we find that it is expelled by the contractions of the bladder, we must cease to increase the number of injections, and to diminish their temperature.—*Ibid.*

